# Three-Dimensional Transperineal Ultrasound Guiding Early Secondary Repair of Obstetric Anal Sphincter Injury in an Incontinent Patient without Suture Dehiscence

**DOI:** 10.3390/diagnostics14010068

**Published:** 2023-12-27

**Authors:** Michele Orsi, Giuseppe Cappuccio, Hayato Kurihara, Gabriele Rossi, Giuseppe Perugino, Enrico Ferrazzi, Carmela Coppola

**Affiliations:** 1 Unit of Obstetrics, Department of Woman, Newborn and Child, Fondazione IRCCS Ca’ Granda Ospedale Maggiore Policlinico, 20122 Milan, Italy; 2 Department of Clinical Sciences and Community Health, Università degli Studi di Milano, 20122 Milan, Italy; 3 Unit of Emergency Surgery, Fondazione IRCCS Ca’ Granda Ospedale Maggiore Policlinico, 20122 Milan, Italy

**Keywords:** pelvic floor disorders, obstetric anal sphincter injury, 3D imaging, ultrasonography, anal incontinence, early secondary repair, multidisciplinary management

## Abstract

We present the case of a 36-year-old primigravida who gave birth to a 3200 g baby by vacuum-assisted (Kiwi OmniCup™) operative vaginal delivery with mediolateral episiotomy. A “y”-shaped perineal tear with a grade IIIC obstetric anal sphincter injury (OASI) was diagnosed and repaired. Two days after delivery, in the absence of suture dehiscence, she started experiencing complete anal incontinence. A decision was made in association with a proctologic surgeon for an early secondary repair. Before surgery, a Three-dimensional transperineal ultrasound (TPUS) was performed. The exam revealed a major defect of the external anal sphincter at the 11 o’clock position. This allowed for the reopening of only a circumscribed area of the perineal suture and repair of the sphincters using the end-to-end technique. The symptoms regressed completely, and follow-up TPUS demonstrated the gradual wound healing process. Anal incontinence, secondary to obstetric anal sphincter injury (OASI), has a severe negative impact on women’s quality of life. TPUS is an effective method to detect sphincter defects and monitor the healing process. This report investigates the feasibility of identifying the sphincter tear in an incontinent puerperal patient without suture dehiscence in order to target early secondary repair while minimizing its extent. TPUS has proven a safe and effective tool to guide early secondary repair of symptomatic OASI complications while minimizing the invasiveness of the procedure. Multidisciplinary management is crucial to ensure the adequate standard of care.

Obstetric anal sphincter injuries (OASIs) complicate approximately 2.1 to 6% of vaginal deliveries [1,2,3,4,5]. OASIs include third-degree tears that involve the anal sphincters, and fourth-degree tears if the rectal mucosa is additionally injured. Further subdivision differentiates grade IIIa and IIIb, involving <50% or >50% of the external anal sphincter (EAS), respectively, and IIIc if extended to the internal anal sphincter (IAS) [6]. OASI is unpredictable. Risk factors have been identified, including primarily operative vaginal delivery, shoulder dystocia, macrosomia, increased maternal body mass index, and nulliparity [1,4,5]. Antenatal perineal massage, warm compresses during the active phase of the second stage of labor, perineal protection at fetal head crowning, and the all/four maternal birthing position have shown a protective effect [7], while the role of episiotomy remains controversial [8,9,10]. OASI carries a risk of developing anal incontinence between 20% and 60% [11,12], with a major negative impact on the quality of life as a result of its physical, psychological, and social implications [13,14]. If anal incontinence persists after primary repair or as a result of tear dehiscence, secondary repair may be indicated [15,16]. While early secondary repair in the case of suture dehiscence has been investigated [16], indications regarding symptomatic cases without suture dehiscence are lacking. In recent years, 3D transperineal ultrasound (TPUS) has been successfully used to diagnose OASIs and monitor the healing process [17], achieving good sensitivity and high specificity in identifying EAS and IAS defects, respectively [18]. It also has the advantages of lower cost, greater availability in gynecological clinics, and higher tolerability [12] than endoanal ultrasound (EAUS), which has been the gold standard so far [14,19].

We present the case of a 36-year-old primigravida with a pregestational BMI of 19. At 38 weeks of gestation, this patient was admitted to our high-risk outpatient clinic for severe polyhydramnios. She underwent induction of labor per protocol with intravaginal dinoprostone. Labor pain was treated using low-dose “walking-epidural” analgesia. After 10 h, she gave birth to a 3200 g baby in anterior right occipito-iliac vertex position with a vacuum (Kiwi OmniCup™, Clinical Innovations, Muray, UT, USA) operative vaginal delivery applied for inadequate maternal pushing efforts with mediolateral episiotomy. After the placental expulsion, the perineum was inspected, and a “y”-shaped laceration beyond the episiotomy involving the external and internal anal sphincter (grade IIIc) was diagnosed. The lesion was repaired with the end-to-end technique for the anal sphincter using Vycril 2/0. Total blood loss was 1300 mL secondary to persistent uterine hypotonia, treated with uterine massage and uterotonics, and bleeding from the perineal wound. Subsequently, an indwelling urinary catheter was placed, and broad-spectrum antibiotic therapy and laxatives were prescribed. On the second postpartum day, following the detection of symptomatic anemia with a hemoglobin of 6.3 g/dL, a decision was made to transfuse the patient with two units of red blood cells. Despite an apparently normally closed perineal suture, a gradual onset of complete anal incontinence for gas and feces occurred from the second day after delivery. A multidisciplinary counselling with a proctologic surgeon was offered to discuss the risks and benefits of early versus delayed anal sphincter repair with the patient. Finally, a decision was made for early secondary repair. Before surgery, a TPUS was performed (Voluson™ S10 Expert, GE Medical Systems, Zipf, Austria). The 3.5–5 MHz ultrasound probe was placed at the anal opening of the patient with 10°–20° inclination toward the ventral direction to locate the anal sphincter [12]. During the examination of the anal sphincter using TPUS, four layers were visualized (from the inner to the outer part): the lumen of the anal canal, the mucous membrane, the IAS hypoechogenic ring, and the EAS hyperechogenic ring (Figure 1). These layers were depicted in a transversal cutting plane. The exam allowed for the identification of a residual defect of the EAS at the 11 o’clock position (Figure 1). At surgery, the suture was reopened for 1 cm in the area indicated using the ultrasound, with confirmation of the sphincter discontinuation; repair using the end-to-end technique with an intermediate resorption of 3/0 was then performed. After the repair, the incontinence regressed completely. The patient was discharged after 3 days. At one-month follow-up, the patient remained asymptomatic and TPUS confirmed the wound healing process (Figure 2).

OASI has a devastating impact on the quality of life of affected women [2,13]. However, in the absence of postpartum suture dehiscence, early sphincter repair is not mandatory. Besides, extensive reoperation has potential risks that may further impact postpartum recovery [15]. The relevance of this case report lies in the potential of TPUS to increase the likelihood of successful early secondary repair while minimizing the area involved in the surgery, thus reducing its invasiveness. To address postpartum anal incontinence, a multidisciplinary approach should be established, including a proctologist and urogynecologist surgeon or urologist in case of associated urinary symptoms, in order to ensure a shared decision-making process and adequate management. In the case we presented, complete anal incontinence was not associated with either urinary symptoms or primary suture dehiscence. However, we believe that this symptom is so disabling that the diagnostic and therapeutic procedure must be immediately activated [18]. Although a gradual spontaneous reduction in symptoms has been reported over the years following delivery, there is consensus that patients with severe impairment are candidates to early repair [20]. The traditional approach to secondary OASI repair was to postpone re-suturing after up to 3 months. This strategy was aimed at allowing for the inflammatory state to regress and any infections to be cured, providing the tissues adequate time to regenerate the blood supply network through a process of neovascularization [16,21]. However, as in our report, some authors reported satisfactory outcomes after early secondary repair, with complication rate comparable to the traditional approach of delayed repair [15,22,23]. We believe this approach should be further explored in future research. With particular reference to severely symptomatic patients, the goal must be to minimize the duration of symptoms with a significant impact on the quality of life, such as anal incontinence, while maintaining a high safety profile of the therapeutic strategy. Intrapartum perineal swelling and postpartum hemorrhage can hinder the clinical diagnosis of OASI immediately after childbirth. In the presented case, the persistent postpartum bleeding may have undermined clinical assessment in the delivery room and favored suboptimal repair. However, in the absence of adequate conditions for optimal surgical repair, delayed repair up to 8 to 12 h has proven equally effective. This choice could also facilitate the involvement of colleagues with higher surgical experience [24]. To overcome these intrapartum challenges in diagnosing OASI, during the last decades, imaging techniques of the anal sphincter have been studied to support the identification and follow-up of postpartum lesions. Although EAUS remains the gold standard, it does not allow for resting sphincter examination, is scarcely available in gynecological clinics, and has a high cost [25]. More recently, TPUS has been demonstrated as an effective alternative method to identifying OASIs and monitor the healing process over time [12,17]. In particular, we believe that all patients symptomatic for postpartum AI should receive this diagnostic assessment, which is less expensive, more available at gynecological clinics, and better tolerated in comparison to EAUS [12]. Furthermore, in this report, we confirmed that TPUS allows for early identification of postpartum residual EAS defect, without the need for waiting for perineal edema resolution [26]. The opportunity of using this minimally invasive diagnostic method should be considered in case of symptomatic patients without suture dehiscence. Therefore, in case of unavailability of this diagnostic technique at the institution where delivery was assisted, we recommend immediate referral of the patient to a center where TPUS available.

In order to minimize a dramatic impact on the patients’ quality of life, it is mandatory to guarantee a rapid and effective diagnostic and therapeutic pathway for women suffering from postpartum anal incontinence. Even in the absence of an evident dehiscence of the primary suture, TPUS has proven a safe and effective tool to guide early secondary repair of symptomatic sequelae of OASI. Preoperative exact localization of the lesion may allow for minimizing the extent of the surgical repair and successfully resolving the symptoms. The multidisciplinary management ensures an adequate standard of care, optimizing the chance of success. Further evidence will provide confirmation of these preliminary findings.

## Figures and Tables

**Figure 1:**
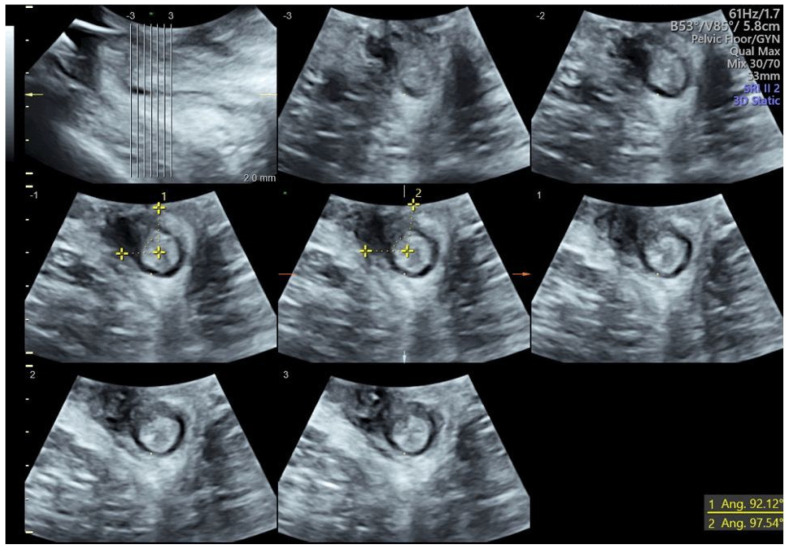
Three-dimensional transperineal ultrasound performed before secondary repair. The angle highlights the external anal sphincter injury at the 11 o’clock position.

**Figure 2:**
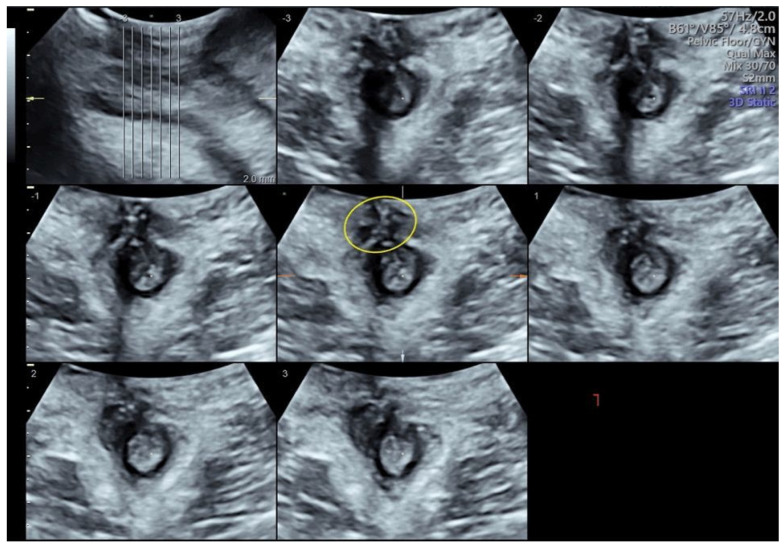
Three-dimensional transperineal ultrasound performed one month after secondary surgery. The anal sphincter repair is indicated by the yellow circle.

## Data Availability

The data analyzed for this study are available upon reasonable request from the referral author.

## References

[1] Hehir M.P., O’Connor H.D., Higgins S., Robson M.S., McAuliffe F.M., Boylan P.C., Malone F.D., Mahony R. (2013). Obstetric Anal Sphincter Injury, Risk Factors and Method of Delivery—An 8-Year Analysis across Two Tertiary Referral Centers. J. Matern.-Fetal Neonatal Med..

[2] Friedman A.M., Ananth C.V., Prendergast E., D’Alton M.E., Wright J.D. (2015). Evaluation of Third-Degree and Fourth-Degree Laceration Rates as Quality Indicators. Obstet. Gynecol..

[3] Ampt A.J., Patterson J.A., Roberts C.L., Ford J.B. (2015). Obstetric Anal Sphincter Injury Rates among Primiparous Women with Different Modes of Vaginal Delivery. Int. J. Gynecol. Obstet..

[4] Meister M.R.L., Cahill A.G., Conner S.N., Woolfolk C.L., Lowder J.L. (2016). Predicting Obstetric Anal Sphincter Injuries in a Modern Obstetric Population. Am. J. Obstet. Gynecol..

[5] Hehir M.P., Rubeo Z., Flood K., Mardy A.H., O’Herlihy C., Boylan P.C., D’Alton M.E. (2018). Anal Sphincter Injury in Vaginal Deliveries Complicated by Shoulder Dystocia. Int. Urogynecol. J..

[6] Cichowski S., Rogers R. (2018). Prevention and Management of Obstetric Lacerations at Vaginal Delivery. Obstet. Gynecol..

[7] Wilson A.N., Homer C.S.E. (2020). Third- and Fourth-degree Tears: A Review of the Current Evidence for Prevention and Management. Aust. N. Z. J. Obs. Gynaecol..

[8] Kapoor D.S., Thakar R., Sultan A.H. (2015). Obstetric Anal Sphincter Injuries: Review of Anatomical Factors and Modifiable Second Stage Interventions. Int. Urogynecol. J..

[9] Frenette P., Crawford S., Schulz J., Ospina M.B. (2019). Impact of Episiotomy During Operative Vaginal Delivery on Obstetrical Anal Sphincter Injuries. J. Obstet. Gynaecol. Can..

[10] Okeahialam N.A., Wong K.W., Jha S., Sultan A.H., Thakar R. (2022). Mediolateral/Lateral Episiotomy with Operative Vaginal Delivery and the Risk Reduction of Obstetric Anal Sphincter Injury (OASI): A Systematic Review and Meta-Analysis. Int. Urogynecol. J..

[11] Sideris M., McCaughey T., Hanrahan J.G., Arroyo-Manzano D., Zamora J., Jha S., Knowles C.H., Thakar R., Chaliha C., Thangaratinam S. (2020). Risk of Obstetric Anal Sphincter Injuries (OASIS) and Anal Incontinence: A Meta-Analysis. Eur. J. Obstet. Gynecol. Reprod. Biol..

[12] Stickelmann A.-L., Kennes L.N., Hölscher M., Graef C., Kupec T., Wittenborn J., Stickeler E., Najjari L. (2022). Obstetric Anal Sphincter Injuries (OASIS): Using Transperineal Ultrasound (TPUS) for Detecting, Visualizing and Monitoring the Healing Process. BMC Women’s Health.

[13] Cotterill N., Norton C., Avery K.N.L., Abrams P., Donovan J.L. (2011). Psychometric Evaluation of a New Patient-Completed Questionnaire for Evaluating Anal Incontinence Symptoms and Impact on Quality of Life: The ICIQ-B. Dis. Colon Rectum.

[14] Carter D., Ram E., Engel T. (2023). Combined 3D Endoanal Ultrasound and Transperineal Ultrasound Improves the Detection of Anal Sphincter Defects. Diagnostics.

[15] Okeahialam N., Thakar R., Kleprlikova H., Taithongchai A., Sultan A. (2020). Early Re-Suturing of Dehisced Obstetric Perineal Wounds: A 13-Year Experience. Eur. J. Obstet. Gynecol. Reprod. Biol..

[16] Okeahialam N.A., Thakar R., Sultan A.H. (2021). Early Secondary Repair of Obstetric Anal Sphincter Injuries (OASIs): Experience and a Review of the Literature. Int. Urogynecol. J..

[17] Ros C., Martínez-Franco E., Wozniak M.M., Cassado J., Santoro G.A., Elías N., López M., Palacio M., Wieczorek A.P., Espuña-Pons M. (2017). Postpartum Two- and Three-Dimensional Ultrasound Evaluation of Anal Sphincter Complex in Women with Obstetric Anal Sphincter Injury. Ultrasound Obs. Gynecol..

[18] Hakim S., Santoso B.I., Djusad S., Moegni F., Surya R., Kurniawan A.P. (2023). Diagnostic Capabilities of Transperineal Ultrasound (TPUS) to Evaluate Anal Sphincter Defect Post Obstetric Anal Sphincter Injury (OASIS)? A Systematic Review. J. Ultrasound.

[19] Volløyhaug I., Taithongchai A., Arendsen L., Van Gruting I., Sultan A.H., Thakar R. (2020). Is Endoanal, Introital or Transperineal Ultrasound Diagnosis of Sphincter Defects More Strongly Associated with Anal Incontinence?. Int. Urogynecol. J..

[20] Barbosa M., Christensen P., Møller-Bek K., Brogaard L., Glavind-Kristensen M. (2021). Can Ultrasound 10 Days after Obstetric Anal Sphincter Injury Predict Anal Incontinence at Long-Term Follow-Up?. Int. Urogynecol. J..

[21] Arona A.J., al-Marayati L., Grimes D.A., Ballard C.A. (1995). Early Secondary Repair of Third- and Fourth-Degree Perineal Lacerations after Outpatient Wound Preparation. Obs. Gynecol..

[22] Soerensen M.M., Bek K.M., Buntzen S., Højberg K.-E., Laurberg S. (2008). Long-Term Outcome of Delayed Primary or Early Secondary Reconstruction of the Anal Sphincter after Obstetrical Injury. Dis. Colon Rectum.

[23] Barbosa M., Glavind-Kristensen M., Christensen P. (2020). Early Secondary Repair of Obstetric Anal Sphincter Injury: Postoperative Complications, Long-Term Functional Outcomes, and Impact on Quality of Life. Tech. Coloproctol..

[24] Nordenstam J., Mellgren A., Altman D., López A., Johansson C., Anzén B., Li Z.-Z., Zetterström J. (2008). Immediate or Delayed Repair of Obstetric Anal Sphincter Tears-a Randomised Controlled Trial. BJOG.

[25] Abdool Z., Sultan A.H., Thakar R. (2012). Ultrasound Imaging of the Anal Sphincter Complex: A Review. BJR.

[26] García-Mejido J.A., Gutiérrez Palomino L., Fernández Palacín A., Sainz-Bueno J.A. (2017). Aplicabilidad de la ecografía transperineal en 3/4D para el diagnóstico de lesiones del esfínter anal durante el posparto inmediato [Applicability of 3/4D transperineal ultrasound for the diagnosis of anal sphincter injury during the immediate pospartum]. Cir. Cir..

